# Supplementary figures and images for: Facile Synthesis of Antimicrobial Aloe Vera-“Smart” Triiodide-PVP Biomaterials
Source: Biomimetics (Basel). 2020 Sep 17;5(3):45. doi: 10.3390/biomimetics5030045 (PMC7558393; doi:10.3390/biomimetics5030045)

## Slide 1
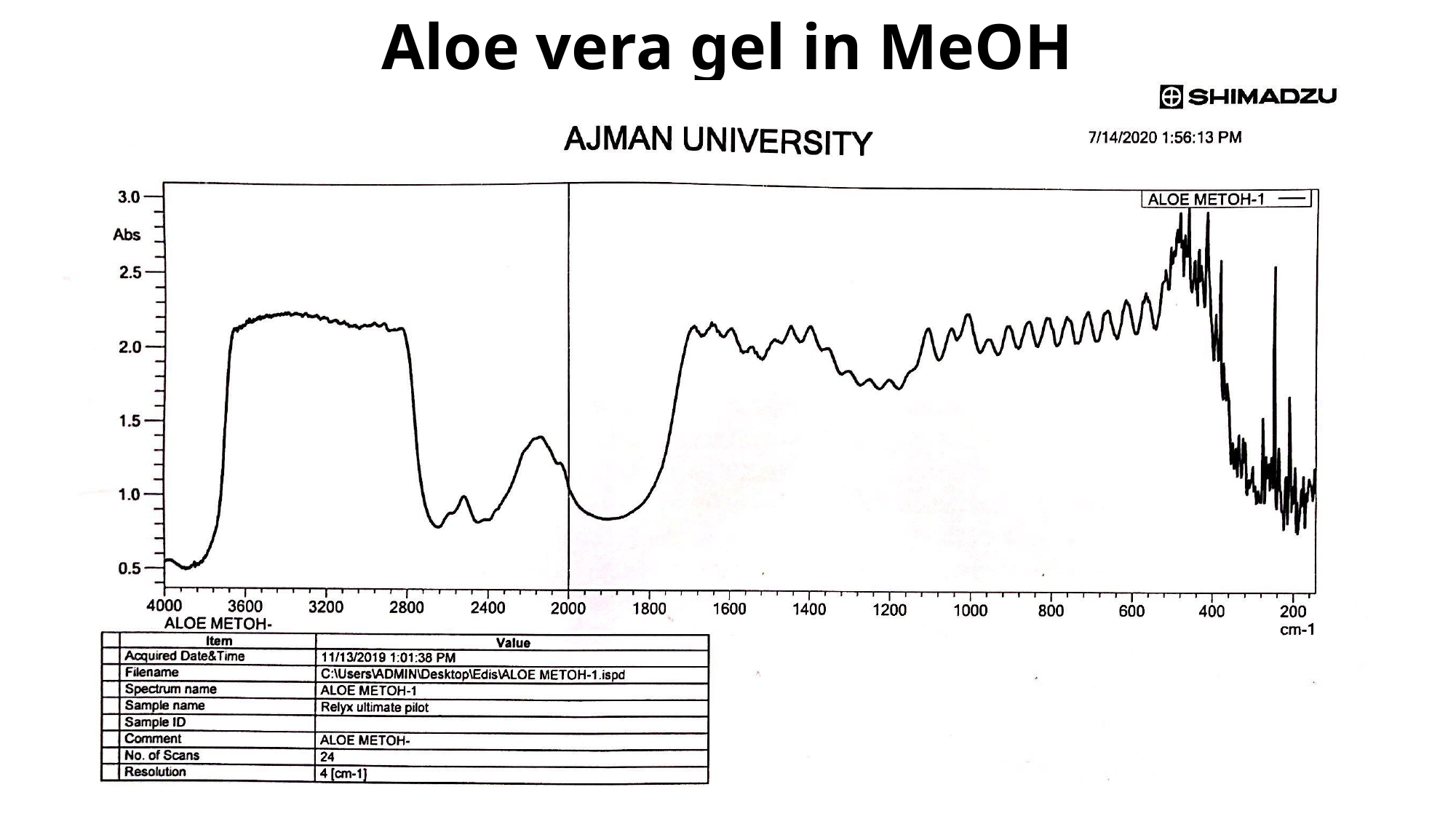

# Aloe vera gel in MeOH

Supplement: Supplementary file 1 [file biomimetics-05-00045-s001.zip › biomimetics-929210-supplementary.pptx]
